# Cooperative effects in differentiation and proliferation between PDGF-BB and matrix derived synthetic peptides in human osteoblasts

**DOI:** 10.1186/1471-2474-12-263

**Published:** 2011-11-21

**Authors:** Thomas Vordemvenne, Jürgen RJ Paletta, Rene Hartensuer, Thomas Pap, Michael J Raschke, Sabine Ochman

**Affiliations:** 1 Department of Trauma, Hand and Reconstructive Surgery, University Hospital Münster, Germany; 2 Department of Orthopedics, University Hospital Marburg, Germany; 3 Institute of Musculoskeletal Research, University Hospital Münster, Germany

## Abstract

**Background:**

Enhancing osteogenic capabilities of bone matrix for the treatment of fractures and segmental defects using growth factors is an active area of research. Recently, synthetic peptides like AC- 100, TP508 or p-15 corresponding to biologically active sequences of matrix proteins have been proven to stimulate bone formation. The platelet-derived growth factor (PDGF) BB has been identified as an important paracrine factor in early bone healing. We hypothesized that the combined use of PDGF-BB with synthetic peptides could result in an increase in proliferation and calcification of osteoblast-like cells.

**Methods:**

Osteoblast-like cell cultures were treated with PDGF and synthetic peptides, singly and as combinations, and compared to non-treated control cell cultures. The cultures were evaluated at days 2, 5, and 10 in terms of cell proliferation, calcification and gene expression of alkaline phosphate, collagen I and osteocalcin.

**Results:**

Experimental findings revealed that the addition of PDGF, p-15 and TP508 and combinations of PDGF/AC-100, PDGF/p-15 and PDGF/TP508 resulted in an increase in proliferating osteoblasts, especially in the first 5 days of cultivation. Proliferation did not significantly differ between single factors and factor combinations (p > 0.05). The onset of calcification in osteoblasts occurred earlier and was more distinct compared to the corresponding control or PDGF stimulation alone. Significant difference was found for the combined use of PDGF/p-15 and PDGF/AC-100 (p < 0.05).

**Conclusions:**

Our findings indicate that PDGF exhibits cooperative effects with synthetic peptides in differentiation and proliferation. These cooperative effects cause a significant early calcification of osteoblast-like cells (p < 0.05). We suggest the combination of synthetic peptides and PDGF as a potential clinical approach for accelerating bone healing or coating osteosynthesis materials.

## Background

Enhancing the osteogenic capabilities of bone matrix for the treatment of fractures and segmental bone defects is an active area of research. It is well-known that the use of oligopeptides or growth factors promotes osteogenic cell proliferation and differentiation in vitro and in vivo [1-4]. There are many different pathways in research to address the acceleration of fracture healing.

The platelet-derived growth factor (PDGF), a paracrine factor in fracture hematoma, has proven its efficacy in early bone healing [5,6]. It is a homodimeric or heterodimeric protein with A and B polypeptide chains that has three possible isoforms: PDGF-AA, PDGF-BB and PDGF-AB [7]. PDGF is a heat-stable, positively charged protein produced by osteoblasts, platelets and monocytes/macrophages. PDGF-B has higher mitogenic and chemotactic potential and also a higher affinity to bone matrix than PDGF-A [8]. Although it is established that bone cells produce and respond to PDGF-B [9], the role of this factor in the healing of fractures has not been fully defined. PDGF-B staining was detected in macrophages and in some primitive mesenchymal cells around the hematoma on day 3 after the fracture. These findings support the hypothesis that PDGF-B is released during platelet degranulation and acts as a paracrine agent [5,10].

With respect to potential coating techniques, PDGF-loaded poly(l-lactide) (PLLA) membranes are used to enhance tissue regeneration [11]. Different release kinetics of PDGF from different materials have been investigated [12] and led to many potential uses of coating materials in periodontal and trauma surgery for implant in-growths or acceleration of bone healing [13,14].

AC-100 (Dentonin™) corresponding with the central sequence of human MEPE (matrix extracellular phosphoprotein or OF45: osteoblast/osteocyt factor 45) is a stimulator for new bone formation by increasing the number of osteoblasts. Cleaved from the SIBLING (Small Integrin-binding LIgand, N-linked Glycoprotein) family, MEPE is secreted in the bone during proliferation and early maturation phases by fully differentiated osteoblasts. It is suggested that the osteogenic activity of AC-100 is mediated by activating integrin signalling pathways in osteoblasts [15]. Furthermore, MEPE has been also shown to promote the formation of reparative dentin [15,16].

The thrombin-related peptide TP508 (Chrysalin™) is a synthetic 23-amino acid peptide representing the natural sequence of the receptor-binding domain of human thrombin (prothrombin amino acids 508-530). This peptide was initially identified by its ability to compete with thrombin for binding to a high-affinity thrombin receptor on fibroblasts and to generate receptor occupancy-dependent mitogenic signals. Subsequently, a number of studies demonstrated a potential therapeutic role of TP508 in tissue repair [17,18]. In fracture repair, TP508 has been shown to promote angiogenesis and enhance bone strength formation by increased induction of early growth factors and inflammatory mediators [19].

p-15 (Pepgen™) is an FDA-approved, tissue-engineered product that contains anorganic bone mineral (ABM) scaffold and is coated with an active peptide sequence. This peptide surface is derived from the α1 chain of collagen Type I and promotes the attachment and proliferation of fibroblasts and osteoblasts to ABM [20,21].

The aim of this study was to test the hypothesis that the combined use of PDGF-BB with synthetic peptides could result in an increase in proliferation and calcification of osteoblast-like cells. This was done by comparing the effects of PDGF and the peptides AC-100, TP508 or p-15 singly or combined in osteoblast-like cells stimulation.

## Methods

### Isolation of human osteoblast-like cells

Bone specimens were obtained from femur neck of patients (age 50-80 years) undergoing total hip replacement according to the guidelines of the local Ethics Committee. Primary osteoblast cultures were obtained from bone specimens using a previously described technique [22] slightly modified for our experiments. Briefly, trabecular bone fragments were cut into pieces, thoroughly rinsed in phosphate buffered saline (PBS) and kept in MEM:HAMS F12 (1:1) (Biowest, Berlin, Germany) medium containing 10% FCS and antibiotics (100 U penicillin/ml, 100 μg streptomycin/ml) (Gibco, Life Technologies). Cultures were initiated in 75-cm^2 ^culture flasks (BD Biosciences, Bedford, MA) within 3 h and incubated at 37°C in a humidified atmosphere of 5% CO_2_. Culture medium was replaced 3 × weekly. Under these conditions, cells migrated out of the bone-forming a monolayer within 3-4 weeks. Primary cell layers were washed in PBS, detached with trypsin EDTA solution (Biowest, Berlin, Germany) and subcultured once in a 1 to 3 ratio. After growth to confluence, cells were released from culture dish as described above. Cells were either frozen at passage 1 or used for experiments in passage 3. Human recombinant PDGF-BB was obtained from R&D Systems, Inc., U.S.A, AC-100 from Acologix, Inc., U.S.A. P-15 and TP508 were synthesized by Sigma-Aldrich, Germany.

### Stimulation of osteoblast-like cells

Cells from ≥ 5 patients were pooled and seeded at a density of 1 × 10^4 ^in 24-well tissue culture plates (Falcon, Becton Dickinson Labware, USA) and chamber slides (Nalge Nunc International Corp., USA). Cells were cultured as described above. In order to gain osteoinductivity, the medium was supplemented with L-ascorbic acid-2-phosphate (0.1 mM) and ß-glycerolphhosphate (10 mM). Stimulation of cultures was done over a period of 2, 5 and 10 days, with the growth factors and peptides listed in Table 1. Cultures without additional growth factors and peptides served as controls.

**Table 1:** Factor concentrations used in the experiments

AC 100	1000 μg/ml
p-15	1000 μg/ml
TP508	500 μg/ml
PDGF - BB	10 μg/ml

### Proliferation and cell number

Growth of cultures was determined cytologically by counting proliferating cells using Ki-67 antibody (Dako Cytomation, Hamburg, Germany) and total cells (methyl green stain). Briefly, after removal of the medium, cells were rinsed twice in PBS, fixed with methanol on the chamber slides and treated with blocking serum followed by incubation with Ki-67 antibody according to the manufacturer's specifications. Detection was done using Vector Stain Kit (Vector Laboratories, Burlingame, USA) and counterstained with methyl green (Vector Laboratories, Burlingame USA). Proliferating cells and total cells were counted in 5 different areas within 3 independent experiments using an Olympus BX51 microscope and Image Pro Plus analysis software (Chromaphor Analysen Technik, Duisburg, Germany).

### Calcification

The degree of mineralization was determined for osteoblast-like cell cultures grown on chamber slides using van Kossa stain. Cells layers were fixed with methanol and incubated in 3% aqueous silver nitrate (30 min). Samples were then washed with deionized water and excess silver was removed using 5% aqueous sodium thiosulphate for approximately 5 min. After rinsing in distilled water, the slices were analyzed histomorphologically using the same microscope and software described above. At least 5 areas (500 μm × 500 μm) in 3 independent experiments were analyzed by 2 independent investigators.

### Total RNA extraction and cDNA synthesis

RNA was extracted from cell layers using RNeasy Mini Kit (Qiagen, Hilden, Germany) according to the manufacturer's specifications and quantified spectrometrically. Starting from 1 μg RNA, 20-μl cDNA was synthesized using Omniscript reverse transcriptase and oligo-dT primer in presence of dNTP (Qiagen, Hilden, Germany).

### Quantitative RT-PCR

Quantitative reverse transcriptase polymerase chain reactions (RT-PCR) were performed and monitored using an ABI Prism 7700 Sequence Detection System (Applied Biosystems, Rotkreuz, Switzerland). The PCR 2× master mix was based on AmpliTAQ Gold DNA-Polymerase (Applied Biosystems, Rotkreuz, Switzerland). Genes of interest were analyzed in cDNA samples (5 μl for a total volume of 25 μl/reaction) using the standard curve method (Perkin Elmer User Bulletin N. 2). Probes were labeled with 6-carboxy fluorescein (FAM) and TAMRA Cycle temperatures and times were as previously described [23]. Primers and probes for human alkaline phosphatase, collagen I, osteocalcin, and 18 s rRNA were previously described and purchased from TIB Biomol, Berlin, Germany [24].

### Statistical Analysis

All values are presented as mean ± standard errors (SE). Statistics were assessed within each of the 3 independent experiments using Mann-Whitney U-test with p < 0.05 considered to be significant.

## Results

### Proliferation

**Figure 1:**
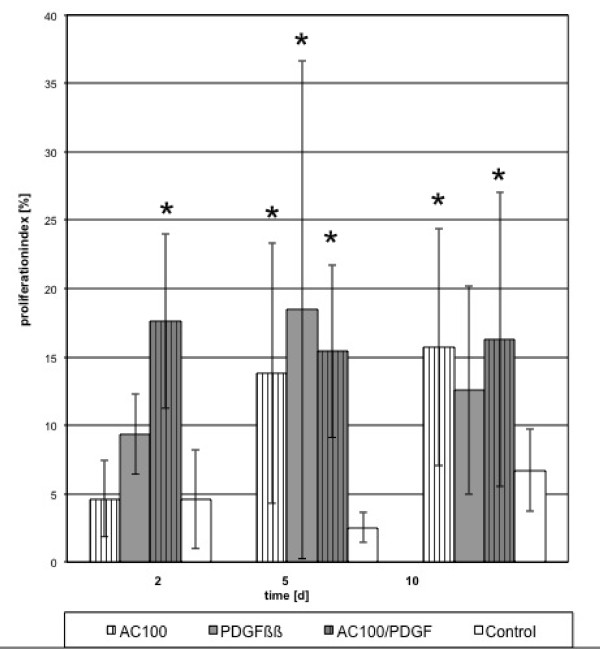
**Proliferation indexed by Ki-67 staining for AC-100 and its combination with PDGF**. Proliferation in the treated cultures was significantly higher than the control at day 5 and 10 (*p < 0.05).

**Figure 2:**
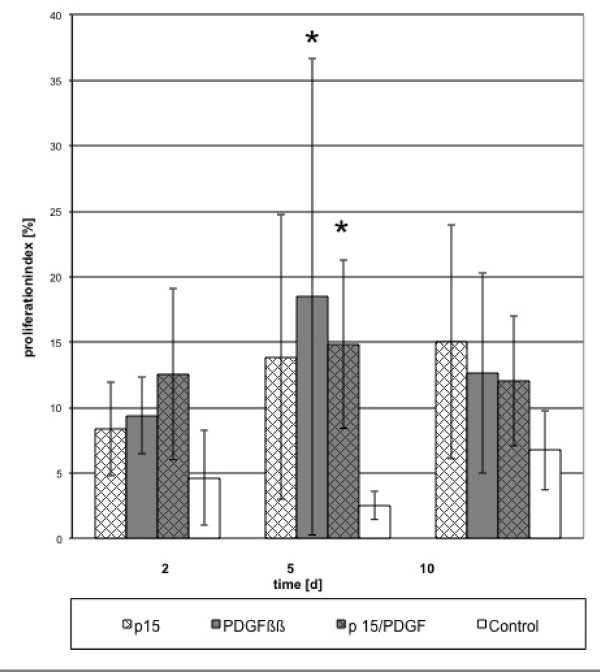
**Proliferation indexed by Ki-67 staining for p-15 and its combination with PDGF**. Proliferation in the treated cultures was significantly higher than the control at day 5 (*p < 0.05).

**Figure 3:**
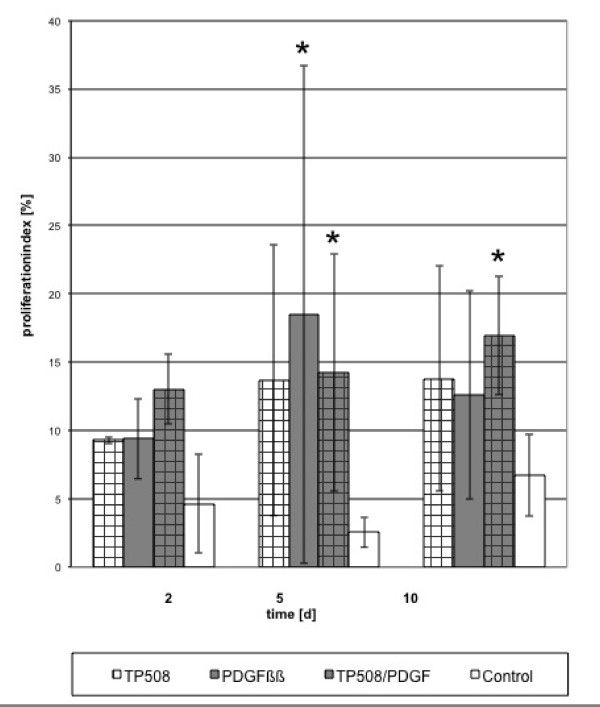
**Proliferation indexed by Ki-67 staining for TP508 and its combination with PDGF**. Proliferation in the treated cultures was significantly higher than the control at day 5 (*p < 0.05).

For the Ki-67 -indexed proliferation, combinations and single factors induced a constant cell proliferation in comparison to the control. Proliferation was significant for all peptides with PDGF (p < 0.05) during the whole time course (Figures 1, 2 and 3).

**Figure 4:**
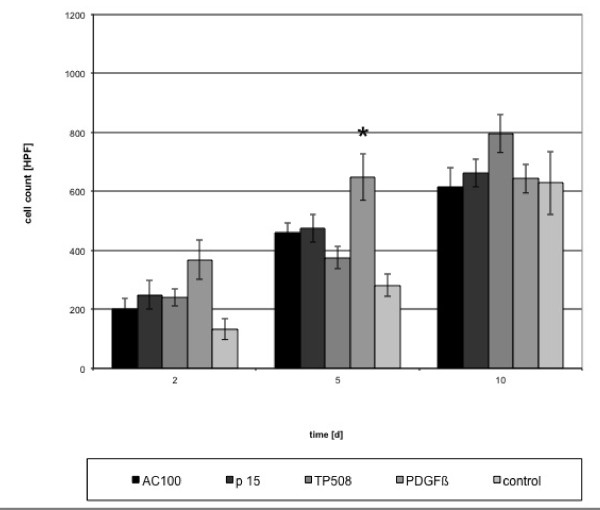
**Absolute cell counts in experiments with single factors**. (HPF = high power field) (*p < 0,001).

**Figure 5:**
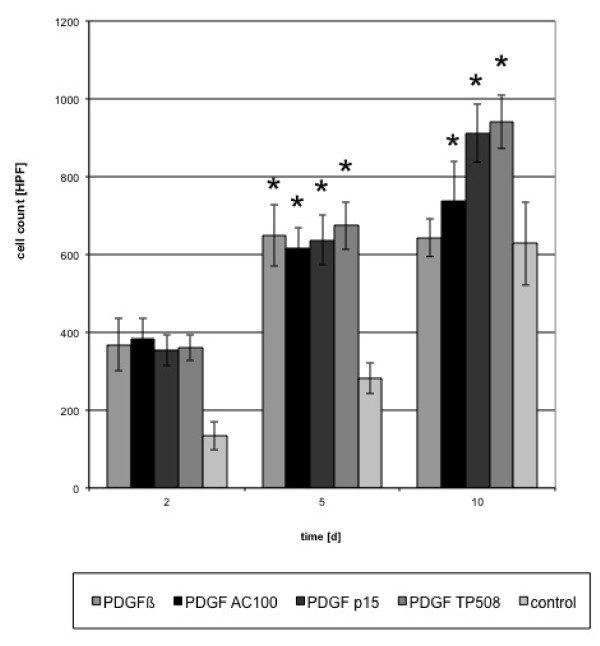
Absolute cell counts in experiments with combined factors; (HPF = high power field) (*p < 0,001).

Figures 4 and 5 show the absolute cell counts calculated from the mean values obtained from 3 independent cultures treated with peptides and PDGF.

In comparison to control, the addition of either PDGF or peptides or their combinations resulted in a significant increase of proliferating cells (p < 0.05), especially in the first 5 days. A significant increase (p < 0.05) was reached at day 10 using PDGF/TP508, PDGF/AC-100 combinations. No significant increase (p > 0.05) was observed when proliferation was compared to cultures induced by either of the single factors.

### Calcification

**Figure 6:**
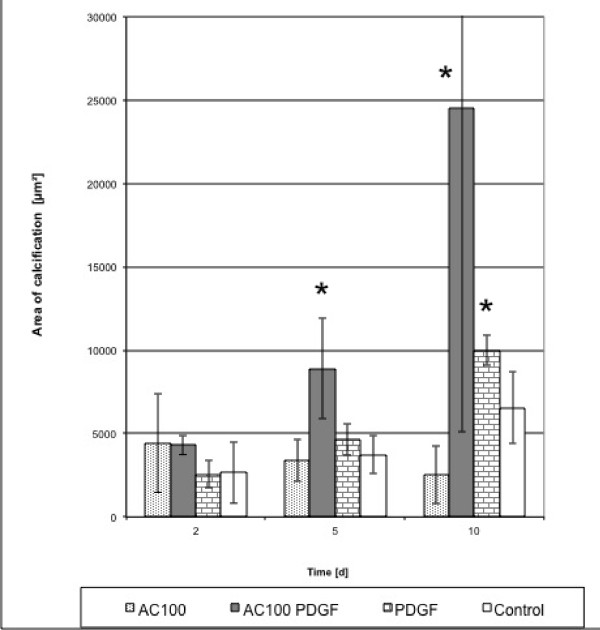
**Calcification indexed by van Kossa staining for AC-100, PDGF, and PDGF/AC-100 combination**. Increase in calcification was significantly higher in PDGF/AC-100 than control at days 5 and 10 (*p < 0.05).

**Figure 7:**
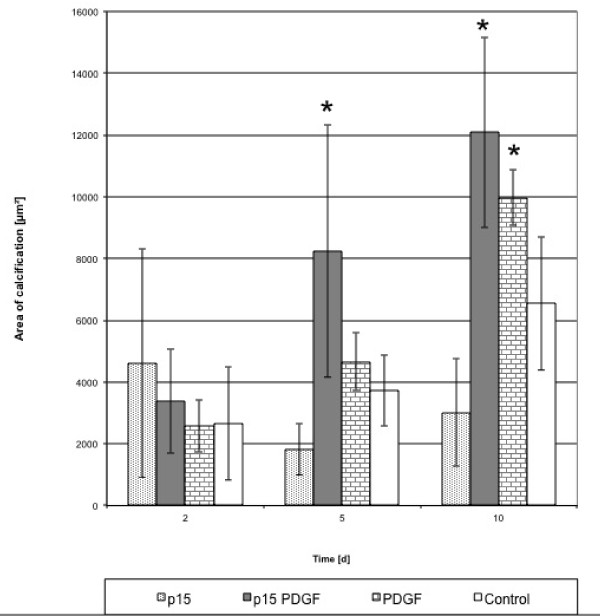
**Calcification indexed by van Kossa staining for p-15, PDGF, and PDGF/p-15 combination**. Increase in calcification was significantly higher in PDGF/p-15 than control at days 5 and 10 (*p < 0.05).

**Figure 8:**
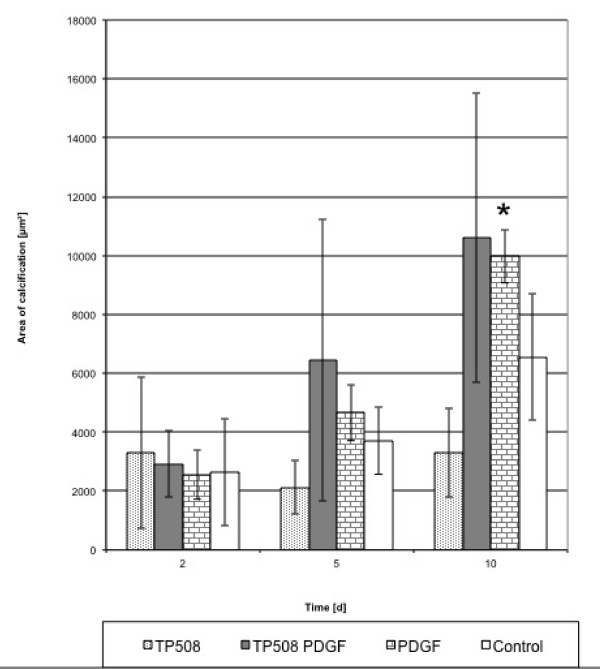
**Calcification indexed by van Kossa staining for TP508, PDGF, and PDGF/TP508 combination**. Increase in calcification was not significantly higher in PDGF/TP508 than control at days 5 and 10 (*p < 0.05).

Although the degree of calcification differed between the single series, an increase of calcification relative to controls was observed in cultures treated with the 3 PDGF combinations. We found that the onset of calcification in osteoblasts occurred earlier and was more distinct compared to the control. This increase in calcification was significantly higher (p < 0.05) relative to the control for PDGF/p-15 and PDGF/AC-100 at days 5 and 10 (Figures 6, 7 and 8).

### Gene expression experiments

#### Alkaline phosphatase

Alkaline phosphatase as a marker of bone formation was expressed in osteoblast-like cells during long-term cultivation, typically showing a maximum of expression at day 5. In our experiments, the expression was unaffected or diminished (not significant at p > 0.05) by addition of AC-100, TP508 and p-15 as well as their combinations with PDGF.

#### Collagen I

With the exception of PDGF treatment, no increase in collagen I expression was observed. A trend of reduced collagen I expression was found, especially with PDGF/AC-100 and PDGF/p-15 combinations but this did not reach statistical significance (p > 0.05).

#### Osteocalcin

Long-term culture of osteoblasts resulted in a time-dependent increase of osteocalcin gene expression, reaching a maximum 10 days after inoculation. In the presence of p-15 and AC-100 as well as PDGF, a 2-fold increase of osteocalcin gene expression was observed in at least in 2 of the 3 independent experiments at 2 and 10 days after inoculation. The combined use of PDGF with either of the matrix-derived peptides had similar effects on the osteocalcin expression but a 2-fold increase in osteocalcin gene expression was observed only in minor experiments. Over all, there were no significant differences between the different cell culture treatments (p > 0.05).

## Discussion

This study investigated the effects of stimulation with PDGF-BB combined with synthetic peptides on osteoblast-like cells, with respect to cell proliferation, calcification and gene expression. In particular, the cooperative action of PDGF with synthetic matrix-derived peptides was explored.

In our experiments, stimulation with peptides exhibited in a significant proliferative effect on the osteoblast-like cells, especially within the early phase of cultivation. The combination of peptides with PDGF resulted in the same proliferative effect, but showed no significant differences when compared to the single substances. The thrombin fragment TP508 enhances proliferation and differentiation and induces chemotaxis in human osteoblasts [25]. AC-100 a synthetic fragment of MEPE, and p-15, the 15 amino acid residue related to the biological active domain or collagen, are known to stimulate proliferation and differentiation of early uncommitted osteoblast precursors [26] and dental pulp stem cells [27]. In literature, AC-100 and TP508 have more proliferative and differentiating effects whereas p-15 is more of a proliferative factor. Our experiments confirm these studies.

Focusing on the differentiation of the osteoblast-like cells, we found that the onset of calcification in osteoblasts under combined stimulation occurred earlier and was significantly higher compared to the corresponding control or PDGF stimulation alone. This calcification was accompanied with an alteration in the gene expression of the matrix components collagen I and osteocalcin in osteoblasts. This may be indicative of a cooperative effect between extracellular matrix (ECM) molecules, peptides and PDGF-BB. It is consistent with observations in other cell types where such effects between integrin, adhesions, growth factors and PDGF have been elucidated [28,29]. Bartold et al. [30] reported that PDGF-BB stimulated the synthesis of proteoglycans. Proteoglycan synthesis precedes collagen synthesis and cell surface proteoglycans can bind a number of growth or cell motility factors [30-32]. The newly formed ECM is an important regulator of cell migration und differentiation. Therefore, the effect of PDGF-BB on collagen synthesis may play an important role in the calcification together with the effect on proteoglycan synthesis. It is reported that the extracellular signal-regulated kinase (ERK) is activated and modulated by PDGF [31,33].

Cell migration studies indicated that PDGF stimulates migration, but has no effect on proliferation. These results were not confirmed by our findings. Wildemann et al. [34] also observed no cell migration under PGDF stimulation released from a drug delivery system but reported an increased proliferation of osteoblasts.

Chaudhary et al. [35] reported that PDGF-BB-treated cells showed very low alkaline phosphatase activity, indicating that PDGF-BB is a potent proliferative factor for osteoblasts but not a factor for differentiation and mineralization [36]. These findings were confirmed by Kono et al. who suggested that the ERK pathway activated by PDGF is a negative regulator of matrix mineralization [37].

Our data gave evidence that the interaction between PDGF and the ECM can be stimulated by biologically active peptides.

As PDGF has been identified as a paracrine factor resulting from the fracture hematoma [38], we speculate that combined use of one of the matrix derived peptides with PDGF-BB is a useful tool to enhance fracture healing or promote healing of osteoarthritis defects.

One limitation of the study maybe the possible interactions between serum in the culture medium and the PDGF-peptide combinations that could modify the over all effects. This should be addressed in future studies.

## Conclusions

In conclusion, our findings support the hypothesis that the combined use of PDGF-BB with synthetic peptides could result in an increase in proliferation and calcification of osteoblast-like cell. We suggest based on our findings that the combined stimulation of osteoblasts with synthetic peptides and PDGF could accelerate bone healing through cooperative effects. These effects have a potential clinical application in coating osteosynthesis materials to accelerate bone healing. However, further studies in vitro and in vivo are warranted.

## Competing interests

The authors disclose financial support for this study by Synthes GmbH, Switzerland. The study was performed in order to identify new factors for a potential support of fracture healing. In detail financial support was given for a assistant medical technician and the biochemical factors used in the study.

## Authors' contributions

All authors contributed to this study. TV made substantial contribution in conception and design of the study, participated in carrying out the experiments, he drafted the manuscript, JRJP carried out the experiments and made the statistical analysis, RH and TP revised critically the manuscript, MJR participate in the design and coordination of the study and gave substantial intellectual support. SO drafted the manuscript, performed the statistical analysis and gave substantial intellectual support. All authors read and approved the final manuscript.

## Pre-publication history

The pre-publication history for this paper can be accessed here:

http://www.biomedcentral.com/1471-2474/12/263/prepub
